# Challenging Case of Marantic Tricuspid Endocarditis Treated With Percutaneous Vegetation Debulking

**DOI:** 10.1016/j.atssr.2022.10.005

**Published:** 2022-10-13

**Authors:** Jessica S. Magarinos, Aswin Mathews, Vladimir Lakhter, Aditi Kalla, Carla Altomare, Sean M. Baskin, Suyog Mokashi

**Affiliations:** 1 Department of General Surgery, Temple University Hospital, Philadelphia, Pennsylvania; 2 Department of Cardiology, Temple University Hospital, Philadelphia, Pennsylvania; 3 Department of Cardiovascular Surgery, Temple University Hospital, Philadelphia, Pennsylvania; 4 Department of Anesthesia, Temple University Hospital, Philadelphia, Pennsylvania

## Abstract

Marantic endocarditis is a rare entity often diagnosed in the setting of advanced malignant disease. We present the case of a 66-year-old man with stage IVb non–small cell lung cancer, with large tricuspid and small aortic valve vegetations. A multidisciplinary team determined the patient to be a poor surgical candidate, but given the likelihood of pulmonary and systemic embolization, an intervention was required. We proposed AngioVac-assisted vegetation debulking of the tricuspid valve to be the best approach in consideration of the small aortic valve vegetation and lung cancer. He successfully underwent this procedure and was discharged on postprocedure day 2.

By the traditional operative indication, tricuspid valve vegetations measuring 2 cm or larger with recalcitrant bacteremia or sepsis are historically considered for surgical procedures. In recent years, tremendous progress has been made in the management of tricuspid valve endocarditis, yet surprisingly few patients are managed with percutaneous approaches. Percutaneous approaches to tricuspid valve endocarditis are not without complications. As a result, the cornerstone of management for tricuspid valve endocarditis is medical therapy. In a less frequently encountered entity, marantic endocarditis or nonbacterial thrombotic endocarditis (NBTE), the main problem is a primary vegetation as a result of platelet thrombi depositions.^1^^,^^2^ Unlike infectious endocarditis, NBTE has a sterile footprint.^1^^,^^2^ The treatment of marantic endocarditis is challenging—patients often have advanced malignant disease, making surgical therapy less appropriate.

We present the case of a 66-year-old man with a past medical history of stage IVb adenocarcinoma of the lung who initially was admitted for worsening dyspnea. He was noted to have an increased oxygen requirement from baseline (2 to 4 L nasal cannula), decreased exercise capacity, and worsening fatigue. His medical history was also significant for deep venous thrombosis, managed with warfarin. His lung cancer, staged IVb, had been treated with carboplatin and pemetrexed for 4 cycles, and then single-agent pembrolizumab was prescribed. He had been observed by a medical oncologist for his malignant disease and was receiving immunotherapy, to which he was noted to be responding. Part of his workup included transthoracic echocardiography (TTE), which revealed a new 1.2 × 2.2-cm small, mobile echogenic mass on the tricuspid valve, with moderate regurgitation. Repeated TTE 1 week later showed mild tricuspid regurgitation, a 16 × 24-mm large, mobile echogenic multilobular pedunculated mass on the posterior leaflet of the tricuspid valve, and a medium fixed echogenic mural mass on the aortic valve (Figure 1). During this time, he remained afebrile, without leukocytosis, and blood cultures were negative. Despite appropriate anticoagulation, the tricuspid valve vegetation had increased in size. Given both left- and right-sided valve involvement in the setting of stage IVb lung adenocarcinoma, the structural heart team was consulted for cross-sector collaboration. Our ability to offer operative intervention was limited by the combination of stage IVb lung adenocarcinoma, poor baseline respiratory status, and reduced capability for wound healing. Throughout all of this evaluation, there still remained the risk of embolization of the tricuspid valve vegetation, resulting in a devastating pulmonary event. The decision was made to pursue AngioVac (AngioDynamics) therapy, a solution that would offer the benefit of vegetation debulking without sternotomy or thoracotomy.

**Figure 1:**
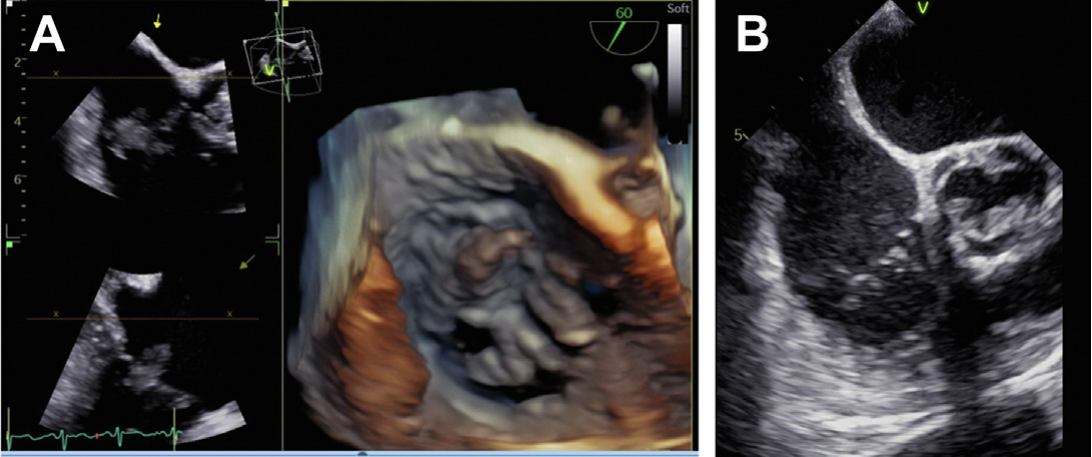
(A) Three-dimensional image of vegetation of the tricuspid valve. (B) Vegetation on tricuspid valve in a modified bicaval view.

The procedure was performed in a hybrid operating room under general anesthesia with transesophageal echocardiography guidance and venovenous extracorporeal membrane oxygenation (ECMO). First, the right femoral vein was accessed with a 6F sheath and upsized to accommodate a return cannula. Next, the right internal jugular vein was accessed and serially dilated to accommodate a 26F Gore DrySeal sheath. Venovenous ECMO was initiated, and the AngioVac was advanced through the right internal jugular vein DrySeal sheath. With use of TEE, the AngioVac was positioned over the vegetations to debulk the vegetations (Figure 2). TEE confirmed a significant reduction in vegetation size (Figure 3). The patient was weaned off ECMO, and the access sites were closed. He was extubated and had an uneventful postprocedure course; he was discharged on postprocedure day 2. The 2-week postprocedure TTE study showed no residual tricuspid vegetations or regurgitation. Unfortunately, the patient ultimately died 3 months after the procedure in a hospice facility.

**Figure 2:**
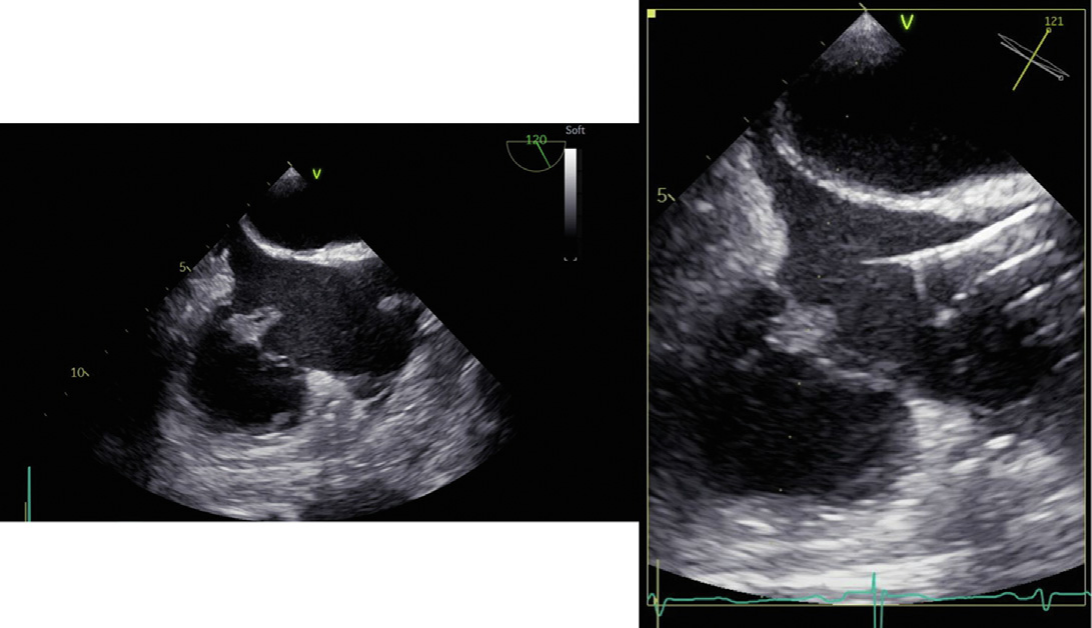
Intraoperative images of AngioVac in use in modified bicaval view.

**Figure 3:**
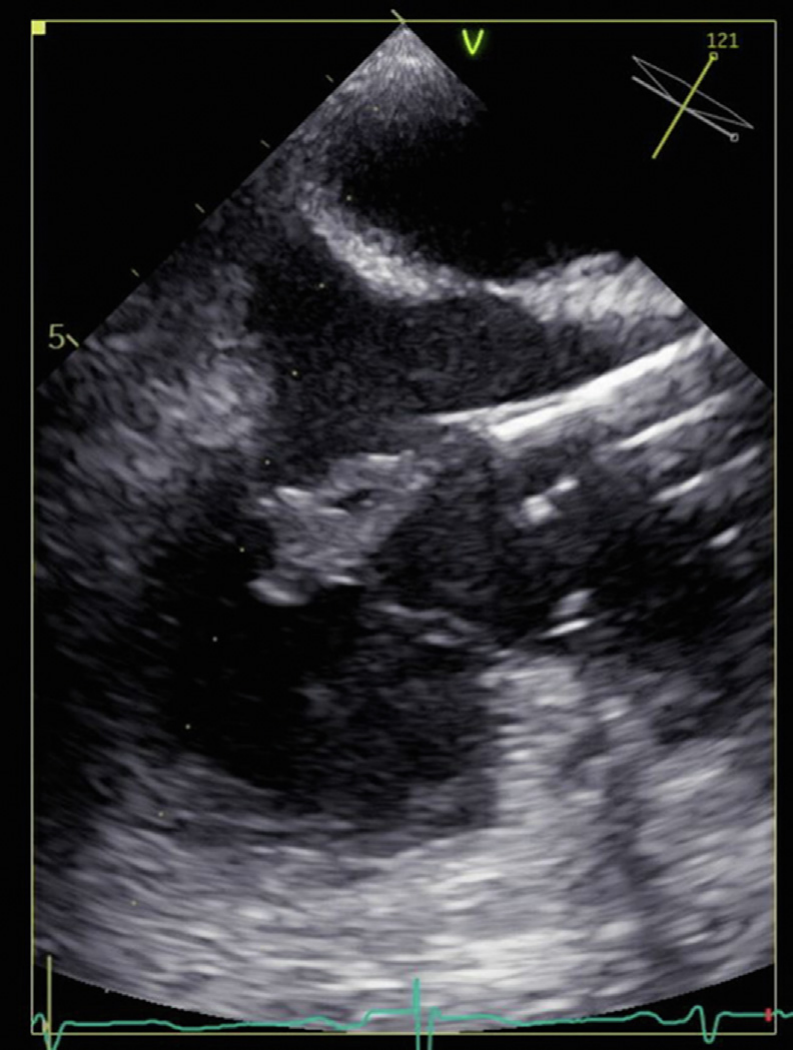
Right ventricular inflow/outflow view after AngioVac procedure showing reduction in vegetation burden.

## Comment

Tricuspid valve endocarditis refractory to antibiotics presents a puzzling clinical challenge. Such challenges might involve patients frequently returning after tricuspid valve operation, sometimes after numerous surgical procedures, with repeated endocarditis. In approaching them, 1 option after maximal medical therapy is to focus on percutaneous therapy, as highlighted in this case.

Marantic endocarditis, a challenging pathologic process, remains a diagnosis of exclusion; more frequently, it is diagnosed during autopsy.^2^ Clinicians must differentiate NBTE from infective endocarditis, primarily to facilitate the appropriate use of antibiotic therapy.^2^ In patients with no obvious infectious source or stigmata of an ongoing infectious process, clinicians should consider NBTE in the differential diagnosis. Echocardiographic imaging is the primary component for workup of this process, with smaller vegetations noted compared with those of infectious origin.^3^

The treatment of NBTE consists of treating the underlying pathologic process.^1^ There is no consensus about the need for anticoagulation in these patients; however, 1 of the end points of treatment of this disorder is to prevent thromboembolic events.^1^^,^^2^ In patients with recurrent thromboembolic events or worsening cardiac function secondary to vegetations, a surgical procedure may be indicated, but risks of the procedure must be carefully discussed. To address that problem, percutaneous treatment options should be explored. The AngioVac procedure, in the setting of infective endocarditis, has been shown to reduce infective burden and to avoid the morbidity of prosthetic valve endocarditis.^4^^,^^5^ To our knowledge, there is no literature discussing the use of AngioVac-assisted debulking in the treatment of NBTE.

The AngioVac venous drainage system has been used in clinical practice since 2012, initially intended for the evacuation of fresh thrombi or emboli from the right atrium and inferior vena cava.^6^ Several studies have reported success rates ranging from 70% to 100% for removal of thrombi with variations secondary to anatomic locations of aspirations.^6^ Several reports have highlighted the clinical effectiveness of off-label uses of AngioVac, such as removal of vegetation from an infected mitral bioprosthesis or free-floating components of a malignant neoplasm.^7^ AngioVac has been proven to have a place in clinical practice for many uses that continue to expand.

NBTE is a complex diagnosis with high associated death. The treatment of this condition is as challenging as the diagnosis, and a surgical procedure is often not a realistic option. The use of innovative procedures such as AngioVac-assisted debulking may offer a low-risk alternative for patients who require intervention.
